# Genome-wide regulation of CpG methylation by ecCEBPα in acute myeloid leukemia

**DOI:** 10.12688/f1000research.28146.1

**Published:** 2021-03-11

**Authors:** Adewale J. Ogunleye, Ekaterina Romanova, Yulia A. Medvedeva

**Affiliations:** 1 Department of Biological and Medical Physics, Moscow Institute of Physics and Technology, Moscow, Russian Federation; 2 Research Center of Biotechnology, Institute of Bioengineering, Russian Academy of Sciences, Moscow, Russian Federation

**Keywords:** Acute myeloid leukemia, Triplex, DNA methylation, long non coding RNA, extra-coding CEBPα

## Abstract

**Background:** Acute myeloid leukemia (AML) is a hematopoietic malignancy characterized by genetic and epigenetic aberrations that alter the differentiation capacity of myeloid progenitor cells. The transcription factor *CEBPα* is frequently mutated in AML patients leading to an increase in DNA methylation in many genomic locations. Previously, it has been shown that *ecCEBPα *(extra coding CEBP *α*) - a lncRNA transcribed in the same direction as *CEBPα* gene - regulates DNA methylation of *CEBPα* promoter in *cis. *Here, we hypothesize that *ecCEBPα* could participate in the regulation of DNA methylation in *trans*.

**Method**: First, we retrieved the methylation profile of AML patients with mutated *CEBPα* locus from The Cancer Genome Atlas (TCGA). We then predicted the *ecCEBPα* secondary structure in order to check the potential of *ecCEBPα* to form triplexes around CpG loci and checked if triplex formation influenced CpG methylation, genome-wide.

**Results:** Using DNA methylation profiles of AML patients with a mutated *CEBPα* locus, we show that *ecCEBPα* could interact with DNA by forming DNA:RNA triple helices and protect regions near its binding sites from global DNA methylation. Further analysis revealed that triplex-forming oligonucleotides in *ecCEBPα* are structurally unpaired supporting the DNA-binding potential of these regions. *ecCEBPα *triplexes supported with the RNA-chromatin co-localization data are located in the promoters of leukemia-linked transcriptional factors such as MLF2.

**Discussion:** Overall, these results suggest a novel regulatory mechanism for *ecCEBPα* as a genome-wide epigenetic modulator through triple-helix formation which may provide a foundation for sequence-specific engineering of RNA for regulating methylation of specific genes.

## Introduction

Acute myeloid leukemia (AML) is a malignant tumor characterized by the proliferation of undifferentiated myeloblasts ^1, 2^. It is the most prevalent form of leukemia in older adults (>60 years) with an annual mortality rate of 50% and a 5-year survival rate of 24% ^2, 3^. With the combined effects of the global increase in average life expectancy and AML drug inefficiency, the number of patients is expected to significantly increase in the coming years ^4, 5^.

The current understanding of the molecular interplay in AML has been defined under two distinct categories; (i) genetic abnormalities and (ii) non-random chromosomal rearrangements. Cases of AML with chromosomal rearrangements as t(15;17) [ *PML-RARA*], t(9;22) [ *BCR-ABL*], inv(16) [ *CBFB-MYH11*], t(8;21) [ *RUNX1-ETO*] are called cytogenetically abnormal (CA-AML), while cases with genetic abnormalities (including frequent mutations in *DNMT3A*, *NPM1*, *CEBPα*, *IDH1/2, TET2, FLT3-ITD*) are called cytogenetically normal (CN-AML) ^1, 4^. The former accounts for 50–55%, while the latter accounts for 45–50% of diagnosed AML cases ^6, 7^. Even though these mutations and chromosomal alterations are crucial for initiating AML, they are not sufficient to explain AML progression, heterogeneity, and relapse ^8^.

Recently, studies have identified the role of non-coding RNAs, especially long non-coding RNAs (lncRNA) in the initiation and progression of cancers ^9– 11^. LncRNAs are emerging functional transcriptional products of at least 200 nucleotides lacking an open reading frame. Although they account for a large proportion of transcriptional products in mammals (about 58,000 loci) ^12^, only a small number of lncRNAs have been well characterized. Although lncRNAs are mostly not conserved evolutionarily, they are heavily regulated suggesting their functional role ^12, 13^. They may function either as signal transducers, protein guides, or molecular scaffolds to regulate transcriptional and epigenetic events ^14– 16^. Some lncRNAs perform these functions in *cis-* by modulating transcription of nearby genes ( *Dum*) ^17^ or act in *trans-*, by modulating genes at multiple distant loci ( *MALAT1*) ^18^, while some can do both ( *HOTAIR*) ^9, 19^.

Recent studies have identified lncRNAs for their remarkable role in regulating major epigenetic processes such as DNA methylation and chromatin remodeling. DNA methylation in mammals is coordinated by one of the three DNA methyl-transferases (DNMT); *DNMT1, DNMT3A,* and *DNMT3B* ^7, 17, 20^. LncRNAs have been identified in recent studies as important agents that can modulate DNA methylation, either by activating or repressing *DNMTs*. For example, the lncRNA *Dum* was discovered to repress a nearby gene *Dppa2* by recruiting multiple *DNMTs* leading to methylation of a promoter region, thus promoting myoblast differentiation ^17^. Conversely, the lncRNA *H19* represses the activity of *DNMT3B* by interacting with *SAHH* which hydrolyzes *SAH*, a step required for *DNMT3B* activation ^21^. *ecCEBPα,* which is transcribed from the *CEBPα* locus, directly blocks *DNMT1* to prevent methylation of proximal and distal located promoters, thus promoting *CEBPα*-mediated granulocyte differentiation ^20^. Mechanistic studies via reduced representation bisulfite sequencing and RNA immunoprecipitation sequencing shows that *ecCEBPα* suppresses DNA methylation in *cis-* by acting as a shield that sequesters *DNMT1* from the *CEBPα* promoter. We speculate that *ecCEBPα* could regulate *DNMT1* activities in distant DNA regions ( *in trans-*) as well. The mechanism of this potential interaction is to be determined.

## Methods

### *ecCEBPα* sequence

In the recent version of GENCODE, *ecCEBPα* is not annotated most likely due to an overlap with a protein-coding gene *CEBPα* on the same strand. We retrieved the complete *ecCEBPα* sequence from the human genome (hg19, chr19: 33298573-33303358) based on information reported in the work of Di Ruscio *et al.* ^20^. *ecCEBPα* is approximately 4.8kb and it overlaps with the intronless *CEBPα* gene (~2.6kb) on the same strand. *ecCEBPα* does not share either the same transcription start site (TSS) or transcription end site (TES) with *CEBPα*, starting ~0.89kb upstream and ending ~1.46kb downstream of the *CEBPα* gene.

### DNA methylation data processing

CpG methylation (Illumina 450K array) and *CEBPα* mutation data for 186 AML patients were retrieved from the Cancer Genome Atlas (TCGA: http://firebrowse.org). CpG methylation levels were measured in 307796 unique loci. We split all the AML patients into two groups based on CEBPα mutation status (13 patients with a CEBPα mutation and 173 patients without a mutation). We classified a CpG position as hypermethylated (HM) in patients with a CEBPα locus mutation if DNA methylation level was significantly increased in the case of a CEBPα mutation (t-test, FDR ≤0.05 and Δ-value ≥0.1) and all non-hypermethylated (NHM) CpG in the case of a CEBPα mutation were classified as non-hypermethylated CpGs (t-test, FDR >0.05 and |Δ-value| <0.1). As a result, we obtained 11955 HM and 261433 NHM CpGs.

### Secondary structure and triplex prediction

As suggested in a previous study ^22^, unpaired RNA nucleotides are more likely to form triplexes with DNA. We predicted RNA secondary structure using RNAplfold (V 2.4.14), from the Vienna suite using a cut-off for pairing probability (-c) of 0.1 ^23, 24^. To search for potential interactions between ecCEBPα and DNA target regions we used only unpaired nucleotides, while the nucleotides predicted to pair were replaced with ‘N’.

DNA target regions were defined as 100 nucleotides centered at each CpG. To predict *ecCEBPα* triplex formation with DNA target regions, we used Triplexator (V 1.3.2) ^25^, since it has higher accuracy of prediction ^14^, with the following optimization parameters suggested in 22: minimum length = 10 nucleotides, error rate = 20%, G-C content = 70%, and filter-repeats = off. Using these parameters out of 307796 unique CpG loci, we predicted 272131 loci with at least one triplex and 35715 without any. Among them, 10351 and 222105 potential triplex targets were predicted in the HM and NHM regions respectively.

To estimate the statistical significance of predicted triplexes we used Triplex domain finder (TDF v 0.12.3), which clusters RNA triplex-forming oligonucleotide (TFO) into DNA binding domains (DBD) ^26^. Briefly, all 272131 CpG loci with at least one predicted triplex were taken as input target regions. By predicting triplexes in the background regions, TDF is capable of estimating the statistical significance of *ecCEBPα* binding between target regions and other non-target CpGs regions. Since TDF allows only to mask regions in the genomic background rather than to select the background explicitly we had to prepare a special mask for the non-target regions. To do so we removed 35715 CpG loci with zero triplex predictions from the human genome using BEDtools subtract (BEDTools v2.29.2). TDF was implemented with a minimum triplex length (-l) of 10 nucleotides, an error rate (-e) of 20%, and (-f) to mask background loci in 100 random samplings (-n).

### RNA:chromatin colocalization analysis

To validate the predicted interactions we used RNA:chromatin interactome obtained with iMARGI method capturing chromatin-associated RNA (caRNA) and their genomic interaction loci ^27^. The data was downloaded from GEO ( GSM3478205). The iMARGI dataset was mapped to the hg38 genome assembly. We used UCSC Liftover to convert *ecCEBPα* sequence coordinates from hg19 to hg38 sequences ^28^. We expanded the DNA coordinates of CpGs by 3.0kb nucleotides upstream and downstream. IntersectBed from BEDTools was used to check the co-location of predicted triplexes and experimentally validated interactions of *ecCEBPα* ^29^. Fisher’s exact test was calculated for the number of confirmed *ecCEBPα* interactions between TDF and iMARGI data.

### GO enrichment analysis

Finally, since Illumina 450K array probes are located close to genes, we performed functional enrichment using BiNGO (v 3.0.3) (binomial test) ^30^ to infer the biological significance of the genes potentially affected by *ecCEBPα* binding.

All statistical analyses were performed using R 4.0 or SciPy v1.5.1 library. Visualization was done in Cytoscape 3.2.0 ^31^ and Python 3.7. Code is available at https://zenodo.org/record/4385259 ^32^.

## Results

### *ecCEBPα* forms triplexes with promoter regions and affects promoter methylation

**Figure 1.**
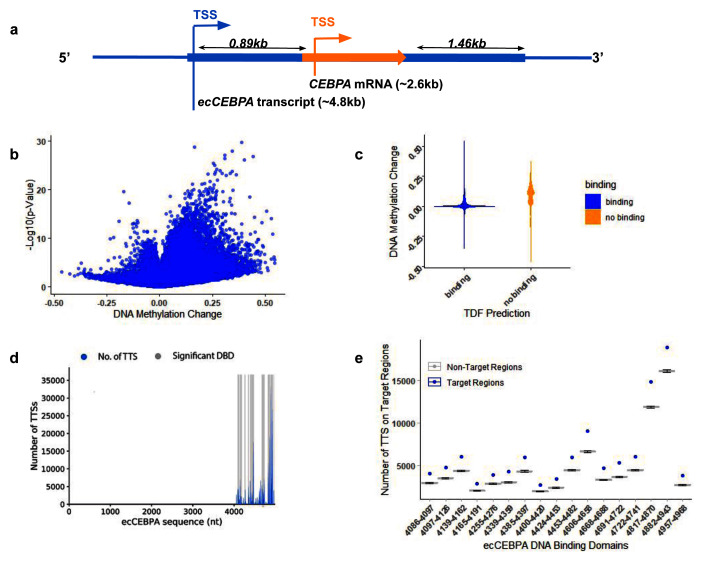
( **a**) Schematic diagram for transcriptional products in the *CEBPα* locus; *CEBPα* is represented by an orange arrow and ec *CEBPα* is represented by the blue line. ( **b**) Global change in DNA methylation ( **c**) Difference in DNA methylation levels between patients with and without *CEBPα* mutation in the regions of *ecCEBPα* predicted binding (non parametric t-test, p-value >1E-10) ( **d**) Number of DNA Triplex Target Site (y-axis) and a location of the corresponding TFO on the *ecCEBPα* (TFO: RNA; x-axis). ( **e**) Number of Triplex Target sites per TFO predicted for NHM and HM CpG regions.

The current study explores the potential of the lncRNA *ecCEBPα* in the modulation of global CpG methylation status in *trans* via direct interaction with DNA regions. *ecCEBPα* (extra coding *CEBPα*), reported in work by Di Russio *et al.* ^20^, is located on chromosome 19 and transcribed from the *CEBPα* locus ( Figure 1a).

Mutations in *CEBPα* locus are a common feature of AML leading to whole genome hypermethylation ( Figure 1b). Since TCGA is focused on protein-coding genes, all reported mutations are located within the *CEBPα* gene and could affect both *CEBPα* and *ecCEBPα.* To investigate if *ecCEBPα* could affect DNA methylation in *trans,* first we checked if it is capable of interaction via forming triple helices (triplexes) with its binding sites and if such interactions affect DNA methylation. We observed that regions capable of forming triplexes with *ecCEBPα* remain protected from global DNA hypermethylation observed in case of a mutation in a *CEBPα* locus ( Figure 1c, Fisher’s exact test, p-value <0.001). This result suggests a negative relationship between DNMT access to promoter sites and *ecCEBPα* binding.

### *ecCEBPα* binding is not affected by the mutation in the *CEBPα* locus

**Figure 2.**
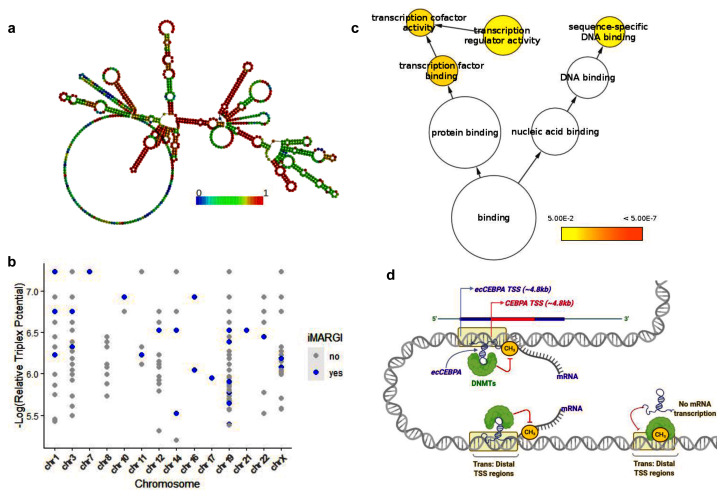
( **a**) Predicted secondary structure of *ecCEBPα.* Nucleotide color represents base pairing probabilities as predicted by RNAplfold. ( **b**) Experimentally validated ecCEBPα triplexes per chromosome. The x-axis represents the chromosome and y-axis represents the triplex potential relative to TTS. Blue points represent all TDF predictions that are present in the iMARGI dataset ( **c**) Functional enrichment of genes located nearby CpG with predicted triplexes. ( **d**) Schematic representation of *ecCEBPα:*DNA interactions in *trans* and its implication on DNA methylation. The presence of *ecCEBPα* inhibits DNA methylation process.

To investigate deeper the potential of ecCEBPα to form triplexes we used Triplex Domain Finder (TDF) - a triplex prediction tool that refines the resolution of predicted TFOs in RNA into DNA binding domains (DBD) and calculates the significance of the number of predicted triplexes for each DBD. Overall, 17 significant DBDs were identified within *ecCEBPα*, interspaced between sequences 4086 and 4968 towards the 3’ end of the lncRNA ( Figure 1d). These DBDs form triplexes with the majority of regions protected from hypermethylation in patients with a *CEBPα* mutation ( Figure 1e, *Extended data:* Supplementary Table 1). The DBD region is located downstream from the *CEBPα* gene suggesting that *ecCEBPα* binding region is not affected by the mutation in the *CEBPα* locus. The predicted secondary structure of the *ecCEBPα* sequence showed that more than 95% of sequence positions from 4087-4987 (~0.5kb from *CEBPα* TES) ( Figure 1d) were unpaired and potentially capable of forming triple helices with the target DNA region ( Figure 2a).

### *ecCEBPα* interacts with predicted binding sites

Since we use relatively relaxed thresholds for triplex prediction, we decided to validate the predicted RNA:DNA triplexes using experimental data obtained with the iMARGI method, allowing detection of RNA-chromatin interactions. We identified 157 *ecCEBPα* contacts within the iMARGI dataset and 29 of them contained predicted triplexes. Altogether, these 29 iMARGI interactions were made up of 182 predicted TTS (Fisher’s exact test, p-value < 2.2E-16) located *in cis and in trans* on 14 chromosomes ( Figure 2b). Chromosomes 19 (the native chromosome for *ecCEBPα*) are accounted for by all predictions.

### Genes protected from methylation by *ecCEBPα* are involved in transcription factor activities

We performed gene ontology analysis on the genes located nearby 182 ecCEBPα triplexes supported by iMARGI. Key gene ontology categories such as nucleic acid binding and transcription factor activities were enriched among putative *ecCEBPα* targets ( Figure 2c). Representative genes among transcription factors include *MLF2, SUV39H2, RBM5, UBTF,* and among sequence-specific DNA binding proteins include *POU2F2, MED12L, and DNASE1L1*. The enrichment in transcription factors (TF) suggests that triplex formation may represent a possible mechanism employed by *ecCEBPα* to regulate TF methylation and as a consequence, their expression.

## Discussion

Unlike other forms of cancers, AML progression is often mutation-independent but may be explained by altered epigenetic regulation, DNA methylation specifically ^1, 8^. In this study, we elucidate a putative mechanism for the regulation of DNA methylation by *ecCEBPα*. In a previous study, *ecCEBPα,* which accompanies the transcription of *CEBPα* on the same locus, was shown to protect the promoter of *CEBPα* from DNA methylation leading to active expression ^20^. We speculated that *ecCEBPα* might perform a similar function in trans. We demonstrated that *ecCEBPα*-DNA triplex formation might provide the molecular basis of this interaction. *ecCEBPα* binding presumably protects the region from genome-wide hypermethylation induced by *CEBPα* mutation in AML patients. *ecCEBPα* contains a TFO/DBD-rich region at its 3’ end, with low pairing probability, suggesting that it is capable of triplex formation. Several of the predicted *ecCEBPα* binding sites, which include transcription factors such as MLF2, SUV39H2, RBM5, UBTF, and sequence-specific DNA binding proteins include *POU2F2, MED12L, and DNASE1L1,* were supported by experimental iMARGI RNA:chromatin interactions data.

Currently, the understanding of lncRNA function and mechanisms of action is limited to a few dozen well-annotated lncRNA transcripts. A few functional characterization attempts are based on the ‘guilt by association’ hypothesis, which may not resonate well with the ability of lncRNA to interact in trans ^33^. As thoroughly reviewed previously, lncRNAs such as *ecCEBPα, Dum, Dali, Dacor1, and LINCRNA-P21* interact with DNA in trans to regulate DNA methylation ^34^. The results presented herein further demonstrate that triplex formation between *ecCEBPα* and CpG containing DNA regions could indeed be regulatory and protect CpG sites from DNMT activity.

Unfortunately, RNA:chromatin interaction protocols are relatively new and the data is available only for a few cell types. Since RNA:chromatin interactions are highly cell-type specific ^35^ and lowly expressed, it is not surprising that we could validate only a few of the predicted interactions. Nevertheless, based on our results, we suggest a model of potential *ecCEBPα* chromatin interaction *in trans* ( Figure 2d). In this model, *ecCEBPα* uses its unpaired regions to directly bind to specific DNA sequences by forming triplexes and in this way prevents DNA methylation in the region of binding. *ecCEBPα* binding to distant regions could be mediated either by 3-dimensional chromatin organization ^17^ which brings them close to *ecCEBPα*.

Recent studies have observed that promoter or transcription start sites (TSS) regions, which tend to be rich in CpG dinucleotides, are TTS-rich and potential triplex-forming hotspots ^36, 37^. Through functional enrichment analysis, we observed that transcription factors might be preferential targets of *ecCEBPα*. Interestingly, previous studies have shown that the suppression of a myeloid leukemia factor ( *MLF2*), an oncogene in breast cancer and myeloid leukemia ^38, 39^ as well as UBTF which controls rDNA expression ^40, 41^ contributes significantly to cancers upon promoter hypermethylation ^40, 42^. The suppressor of variegation 3-9 homolog 2 ( *SUV39H2*), a histone-lysine-N-methyltransferase which regulates the hypermethylation H3K9 has also been reported to indirectly influence over 450 promoters in AML ^43^. Having in mind that *ecCEBPα* is transcribed from CEBPα locus - a key transcription factor of hematopoiesis - this lncRNA could participate in the formation of a hub in the hematopoiesis regulatory network.

## Conclusion

In conclusion, we have shown that ecCEBPα could serve as a trans-acting regulatory agent protecting its binding sites from genome-wide CpG methylation, and its dysregulation could contribute to aberrant methylation profile in AML patients. These results suggest a novel regulatory mechanism for ecCEBPα as a modulator of DNA methylation through triplex formation providing a foundation for sequence-specific engineering of RNA for regulating methylation of specific genes.

## Data availability

### Underlying data

Complete ecCEBPα sequence retrieved from the human genome (hg19, chr19: 33298573-33303358): https://www.ncbi.nlm.nih.gov/assembly/GCF_000001405.13/

*CEBPα* mutation data for 186 AML patients retrieved from the Cancer Genome Atlas (TCGA): http://firebrowse.org.

GEO: Embryonic kidney that expresses SV40 large T antigen, Accession number GSM3478205: https://www.ncbi.nlm.nih.gov/geo/query/acc.cgi?acc=GSM3478205

Zenodo: josoga2/eccebp-alpha-project: F1000 Code Release, https://doi.org/10.5281/zenodo.4385259 ^32^

This project contains the following underlying data:

- DNA_BINDING_DOMAINS_ID.tsv
- predicted_secondary_structure_of_ecCEBPA.fa
- probes.csv (main data)

Code used for analysis available from: https://github.com/josoga2/eccebp-alpha-project/tree/f1000

Archived code as at time of publication: https://doi.org/10.5281/zenodo.4385259 ^32^

### Extended data

Zenodo: Supplementary Data for Secondary structure and DNA binding domain prediction, http://doi.org/10.5281/zenodo.4433222 ^44^.

This project contains the following extended data:

- Supplementary Table 1: Summary table of DNA binding domains (DBD), the counts of target regions within the genome and statistical analysis.
- ecCEBPα secondary structure prediction with RNAplfold

Data and code are available under the terms of the Creative Commons Attribution 4.0 International license (CC-BY 4.0).
