# Supplementary material for: Genome-wide regulation of CpG methylation by ecCEBPα in acute myeloid leukemia
Source: F1000Res. 2021 Aug 31;10:204. Originally published 2021 Mar 11. [Version 2] doi: 10.12688/f1000research.28146.2 (PMC8444155; doi:10.12688/f1000research.28146.2)
Supplement: Peer review history

# Peer review: Reviewer response for version 2

**DOI:** 10.5256/f1000research.65190.r93095

**Published:** 2021-09-15

**Authors:** Amrita Singh

**Affiliations:** 1 CSIR Institute of Genomics and Integrative Biology (CSIR-IGIB), New Delhi, Delhi, India

All the major comments raised by the reviewer have been answered thoroughly by the authors. The additional methylation analysis of HL-60 datasets in both normal and overexpressed ecCEPBα is highly appreciated. Moreover, re-labelling of the plots as highlighted previously has made it easier to comprehend them. However, there are few minor technical remarks, both figures 1 and 2 do not have the legend for the updated figure 1(d) and 2 (c), and accordingly, the subsequent legends labelling needs to be modified.

Is the work clearly and accurately presented and does it cite the current literature?

Partly

If applicable, is the statistical analysis and its interpretation appropriate?

I cannot comment. A qualified statistician is required.

Are all the source data underlying the results available to ensure full reproducibility?

Yes

Is the study design appropriate and is the work technically sound?

Yes

Are the conclusions drawn adequately supported by the results?

Partly

Are sufficient details of methods and analysis provided to allow replication by others?

Partly

Reviewer Expertise:

Structure function relationship of noncoding RNA, with major focus on LncRNA function via triple helical structures.

I confirm that I have read this submission and believe that I have an appropriate level of expertise to confirm that it is of an acceptable scientific standard.

# Peer review: Reviewer response for version 1

**DOI:** 10.5256/f1000research.31133.r81320

**Published:** 2021-06-10

**Authors:** Oleg N. Demidov

**Affiliations:** 1 INSERM U1231, Faculty of Medicine and Pharmacy, University of Bourgogne-Franche Comté, Dijon, France

The manuscript by Ogunleye AF and co-authors describes a novel phenomenon of lncRNA ecCEBPα -dependent regulation of methylation in several genes. The authors analyzed the open datasets and showed that mutations in CEBPA gene (coding both, CEBPA transcriptional factor and lncRNA ecCEBPα) changed the global CpG methylation status in AML cells. The manuscript is nicely written, conclusions are logically justified. This work will be interesting to the readers in the epigenetic regulation and cancer research field.

 Still, I have several concerns about the analysis. There are a lot of predicted triplex target regions for ecCEBPA genome-wide. Some of them could be false-positive predictions. Is there any way to confirm the observed effect only of experimentally validated contacts? Or in regions where predictions are most reliable?

 Also, it is unclear other genes, not only transcription factors, predicted to be regulated by ecCEBPA are related to hematopoiesis. It would be helpful to have at least some hypotheses in the discussion. If there is some link found between ecCEBPA targets and regulation of hematopoiesis, it would really strengthen the conclusions of the work. Probably, KEGG pathway enrichment analysis may help with that.

**Minor comments:**

 It is unclear on the figure with a secondary structure of ecCEBPA where the triplex-forming region is located.

Is the work clearly and accurately presented and does it cite the current literature?

Yes

If applicable, is the statistical analysis and its interpretation appropriate?

Yes

Are all the source data underlying the results available to ensure full reproducibility?

Yes

Is the study design appropriate and is the work technically sound?

Yes

Are the conclusions drawn adequately supported by the results?

Yes

Are sufficient details of methods and analysis provided to allow replication by others?

Yes

Reviewer Expertise:

Models of human diseases, cancer research, fibrosis, paging

I confirm that I have read this submission and believe that I have an appropriate level of expertise to confirm that it is of an acceptable scientific standard.

# Response

**Published:** 2021-08-05

**Authors:** ADEWALE OGUNLEYE

**Affiliations:** Moscow Institute of Physics and Technology, Russian Federation

**Response to Reviewer Two**

** Comment one:** There are a lot of predicted triplex target regions for ecCEBPA genome-wide. Some of them could be false-positive predictions. Is there any way to confirm the observed effect only of experimentally validated contacts? Or in regions where predictions are most reliable?

**Response one**: We are grateful to the reviewer for this suggestion. We performed a validation for the most reliable predicted triplexes using only experimentally validated ecCEBPA:DNA contacts. Overall, a mean methylation delta score of only 0.01 (p-value < 1E-09) was observed which further emphasizes the protective effect of ecCEBPA. 

 The following text and images were added to the main body of the paper:

*“Since these ecCEBPA contacts have been experimentally validated, we suggested that they are the most reliable regions where ecCEBPA might have a protective effect from DNA methylation. Overall, a mean methylation delta score of experimentally validated binding sites is significantly lower than that of the non-binding sites (p-value < 1E-09) (Figure 2C).”*

 New figure panels (1&2) here: https://drive.google.com/file/d/1iynm-t0DfGkxLF5vsumG4iCcfo2u2xCJ/view?usp=sharing 

**Comment two**: Also, it is unclear if other genes, not only transcription factors, predicted to be regulated by ecCEBPA are related to hematopoiesis. It would be helpful to have at least some hypotheses in the discussion. If there is some link found between ecCEBPA targets and regulation of hematopoiesis, it would really strengthen the conclusions of the work. Probably, KEGG pathway enrichment analysis may help with that.

**Response two**: We thank the reviewer for this important comment. Indeed, we discovered that other non-transcription factor genes have links to acute myeloid leukemia via literature mining. Unfortunately, this could not be enriched through KEGG. We suggest including a supplementary table that details the different roles of the identified genes.

 In the main text, we added:

*“Out of the 33 genes we identified to be targets of ecCEBPA, 16 genes (ICAM1, PDXK, etc.) are clearly related to hematopoiesis and various leukemias. A summary of the genes and their function is provided in Table 1.”*

**Table 1: **AML specific function of nearby genes that are protected by ecCEBPA (added to the paper).

# Peer review: Reviewer response for version 1

**DOI:** 10.5256/f1000research.31133.r81314

**Published:** 2021-04-15

**Authors:** Amrita Singh

**Affiliations:** 1 CSIR Institute of Genomics and Integrative Biology (CSIR-IGIB), New Delhi, Delhi, India

The article has addressed an important question of the ability of a lncRNA to act in trans and regulate a multitude of genes, which might be affecting the progression of acute myeloid leukemia. The authors have also highlighted the possibility of a triplex structure for defining the binding of lncRNA at its target gene CpG region. However, there are certain parts wherein the authors haven't been able to convey their points properly.

1. In Figure 1(b), a global DNA hypermethylation has been represented in AML patients with CEBPα mutation, but the numbers of the HM and NHM sites in the methods section do not reflect the same. It shows higher sites for NHM, than the HM sites, kindly check if some mislabeling is there at the authors part.
2. Also, the reviewer has failed to understand Figure 1(c). In both the x-axis and the side legend, binding and non-binding have been depicted, but which one is the AML patients with and without mutation isn't clear from the figure. 
3. There are few other key points that were not clear;
  (a) what is the level of ecCEBPα expression in AML patients with and without mutation.
  (b) Additionally, if the expression of the lncRNA remains the same in both cases, how does one explain the increase in binding of the lncRNA and subsequent higher NHM sites, in the case of AML patients with CEBPα mutation?
  (c) Moreover, a previous report (Di Ruscio *et.al ^1^)* on ecCEBPα had also analyzed genome-wide methylation, in which they report that methylation levels remain unchanged even when ecCEBPα was overexpressed. This is in contrast with the major theme of the paper i.e., methylation of genes in trans is affected by the ecCEBPα. The authors should comment on this in the discussion part. 

Is the work clearly and accurately presented and does it cite the current literature?

Partly

If applicable, is the statistical analysis and its interpretation appropriate?

I cannot comment. A qualified statistician is required.

Are all the source data underlying the results available to ensure full reproducibility?

Yes

Is the study design appropriate and is the work technically sound?

Yes

Are the conclusions drawn adequately supported by the results?

Partly

Are sufficient details of methods and analysis provided to allow replication by others?

Partly

Reviewer Expertise:

Structure function relationship of noncoding RNA, with major focus on LncRNA function via triple helical structures.

I confirm that I have read this submission and believe that I have an appropriate level of expertise to confirm that it is of an acceptable scientific standard, however I have significant reservations, as outlined above.

## References

[1] : DNMT1-interacting RNAs block gene-specific DNA methylation.*Nature*.2013;503(7476) : 10.1038/nature12598371-610.1038/nature1259824107992PMC3870304

# Response

**Published:** 2021-08-05

**Authors:** ADEWALE OGUNLEYE

**Affiliations:** Moscow Institute of Physics and Technology, Russian Federation

**Response to Reviewer One**

**Comment One:** In Figure 1(b), a global DNA hypermethylation has been represented in AML patients with CEBPα mutation, but the numbers of the HM and NHM sites in the methods section do not reflect the same. It shows higher sites for NHM, than the HM sites, kindly check if some mislabeling is there at the authors part.

**Response Two:** We believe that there is a confusion here. The plot was correct and reflected the change in DNA methylation in all CpG positions including those that were not statistically differentially methylated. We counted hypermethylated CpG (t-test, FDR ≤0.05 and Δ-value ≥0.1) and those that were not hypermethylated (t-test, FDR >0.05 and |Δ-value| <0.1). In this way, we refer to non-hypermethylated (NHM) to any CpG with DNA methylation change less than 0.1, which includes either not changing or hypomethylated and not only hypomethylated. To avoid this confusion we suggest replacing the plot with the one below. We specified a 0.1 mid-point to aid visualization. (Figure 1b).

**Comment Two: **Also, the reviewer has failed to understand Figure 1(c). In both the x-axis and the side legend, binding and non-binding have been depicted, but which one is the AML patients with and without mutation isn't clear from the figure.

**Response Two:** Each violin in Figure 1(c) is a representation of the differential methylation score between CpGs of AML patients with and without CEBPa mutation. The blue violin represents all CpGs that are located in ecCEBPA binding sites, while the orange violin represents CpGs that do not bind with ecCEBPA. To provide clarity, we corrected the labels on the X and Y axes and added explicit explanation in the legend of the figure (Figure 1c).

 Difference in methylation = Mean methylation of CpGs in AML patients with CEBPA mutation - Mean methylation of CpGs in AML patients without CEBPA mutation.

**Comment 3: **There are few other key points that were not clear;

 (a) what is the level of ecCEBPα expression in AML patients with and without mutation.

 (b) Additionally, if the expression of the lncRNA remains the same in both cases, how does one explain the increase in binding of the lncRNA and subsequent higher NHM sites, in the case of AML patients with CEBPα mutation?

 (c) Moreover, a previous report (Di Ruscio et.al1) on ecCEBPα had also analyzed genome-wide methylation, in which they report that methylation levels remain unchanged even when ecCEBPα was overexpressed. This is in contrast with the major theme of the paper i.e., methylation of genes in trans is affected by the ecCEBPα. The authors should comment on this in the discussion part. 

**Response:**

**(a&b): **It is relatively difficult to estimate the expression level of ecCEBPA since this gene is not in the annotation used by TCGA. The raw data is not freely available in TCGA. ecCEBPA gene also overlaps with the CEBPA gene making the estimation of expression of each of the genes even more complicated. Since the mutation in AML patients happens in the body of CEBPA gene, we do not expect a significant change in the expression of either CEBPA or ecCEBPA. 

 We explained the relationship between ecCEBPA binding and methylation in the first result section (Figures 1c,d&e). We suggested that the mutation(s) in CEBPA does not affect the expression of the lncRNA, but rather the ability to bind its targets. 

 We further emphasize this point in the paper with this line (discussion):

*“Di Ruscio et al. confirmed that most mutations in CEBPα do not influence the expression of ecCEBPα but rather, its ability of the RNA to fold properly. We suggest that the inability to fold properly even in overexpressed cases may affect its structural ability to bind its targets.”*

**(c)**: We are very grateful to the reviewer for this valuable comment. In response to this, we retrieved the DNA methylation data (RRBS) for wild-type HL-60 cells ( ENCODE Dataset) and overexpressed HL-60 cells ( Di Ruscio *et al* ). We then calculated the methylation difference between the two cell states and compared it between the ecCEBPA binding sites versus the non-binding sites. Non ecCEBPA binding sites were more methylated in comparison to ecCEBPA binding sites (Fisher Test: p-val = 1.24E-09). Bearing in mind that ecCEBPA targets are located genome-wide, it is tempting to suggest that “enforced overexpression” (which was achieved with the R1 variant of ecCEBPA; comprise of downstream ecCEBPA sequences) of ecCEBPA strongly protects local CpGs from methylation while the rest of the genome gain some methylation. 

 We added the following statement to the results in the main text:

*"Furthermore, the overexpression of ecCEBPA in HL-60 cells (Wang et al) lead to ecCEBPA binding sites stay unmethylated while non ecCEBPA binding sites gain methylation (Fisher Test: p-val = 1.24E-09) , suggesting a protective effect on ecCEBPA’s binding to its target locations (Fig 1d)."*
